# Glucocorticoid-induced adrenal insufficiency: physiological dose tapering promotes recovery

**DOI:** 10.1530/EC-25-0625

**Published:** 2026-01-13

**Authors:** Rajeev Mehta, Katharine Lazarus, Angelica Sharma, Pei Chia Eng, Kavita Narula, Sirazum Choudhury, Deborah Papadopoulou, Zin Htut, Tricia M-M Tan, Karim Meeran

**Affiliations:** ^1^ Division of Endocrinology and Investigative Medicine, Imperial College London, London, UK; ^2^ Department of Endocrinology, Imperial College Healthcare NHS Trust, London, UK; ^3^ Department of Endocrinology, National University Hospital, Singapore; ^4^ Department of Clinical Biochemistry, Northwest London Pathology, London, UK

**Keywords:** glucocorticoid-induced adrenal insufficiency, hypothalamic–pituitary–adrenal axis, cortisol, prednisolone, prednisone, tapering

## Abstract

**Objective:**

Glucocorticoid discontinuation is complicated by glucocorticoid-induced adrenal insufficiency. Guidelines discourage tapering below physiological doses (prednisolone 3–6 mg) when morning cortisol is ≤300 nmol/L, with values <150 nmol/L thought to indicate persistent adrenal insufficiency, although this may underestimate hypothalamic–pituitary–adrenal axis suppression from such doses. We aim to evaluate how hypothalamic–pituitary–adrenal axis function evolves during physiological dose tapering and assess whether current cortisol thresholds restrict successful discontinuation.

**Design:**

This is a retrospective cohort study.

**Methods:**

Adults (*n* = 65) with long-term glucocorticoid use for inflammatory disease undergoing prednisolone tapering between 2019 and 2024 were included. Serial short Synacthen tests (*n* = 52) on reducing prednisolone doses (≤5 mg) were analysed using linear mixed-effects modelling. Nadir morning cortisol values at doses ≤5 mg from successful weans were compared with guideline thresholds.

**Results:**

At referral, the mean age was 55.4 ± 16.4 years, with median prednisolone dose and duration of therapy being 5 (3.5–5) mg and 23 (6.5–66.5) months, respectively. For each 1 mg dose reduction, morning and post-Synacthen cortisol rose by 48.8 nmol/L and 57.5 nmol/L (both *P* < 0.001), respectively, with reductions >2 mg producing larger cortisol increases than 1 mg reductions (both *P* < 0.05). Among completed wean attempts (*n* = 47), 81% (*n* = 38) were successful. Of these, 42% (*n* = 16) had a nadir morning cortisol <150 nmol/L, including six with values <28 nmol/L. No adrenal crises occurred.

**Conclusions:**

Physiological dose tapering in glucocorticoid-induced adrenal insufficiency enables, rather than follows, hypothalamic–pituitary–adrenal axis recovery, with structured, symptom-led tapering being safe and effective. Future guidelines should recognise that the HPA axis is suppressed by physiological doses.

**Significance statement:**

In this retrospective cohort study evaluating 65 adults with long-term glucocorticoid use for inflammatory disease undergoing prednisolone tapering, each 1 mg dose reduction from a maximum starting dose of 5 mg increased morning and post-Synacthen cortisol by 48.8 nmol/L and 57.5 nmol/L (both *P* < 0.001), respectively, with reductions >2 mg producing larger cortisol increases than 1 mg reductions (both *P* < 0.05). Furthermore, sixteen patients with a nadir morning cortisol <150 nmol/L, including six with values <28 nmol/L, were able to safely and successfully discontinue prednisolone. These findings have important implications for the management of glucocorticoid-induced adrenal insufficiency, with hypothalamic–pituitary–adrenal axis recovery driven by physiological dose reduction itself, and successful tapering enabling rather than following axis recovery. The routine use of short Synacthen tests in glucocorticoid-induced adrenal insufficiency is not supported by this study.

## Introduction

Glucocorticoids (GCs) are widely used in the management of autoimmune and inflammatory diseases (1), with prednisolone the most commonly prescribed oral GC for chronic use (2, 3). However, prolonged GC exposure is associated with significant adverse effects, including osteoporosis (4), type 2 diabetes mellitus (T2DM) (5), increased cardiovascular (6) and infection (7, 8) risk, and increased mortality (9). The growing availability of biologic therapies has enabled patients to achieve inflammatory disease control with reduced reliance on long-term GC therapy (10), making safe GC discontinuation an increasingly important clinical goal. Yet, discontinuation is complicated by the risk of GC-induced adrenal insufficiency (GC-AI). This is among the most common forms of adrenal insufficiency (AI) (11), caused by GC-mediated suppression of the hypothalamic–pituitary–adrenal (HPA) axis through negative feedback on corticotropin-releasing hormone and adrenocorticotropic hormone (ACTH) release, leading to corticotroph and adrenal atrophy.

The European Society of Endocrinology (ESE) and Endocrine Society (ES) advise weaning patients to a physiological GC dose prior to evaluating the HPA axis (12). It defines a ‘physiological equivalent’ prednisolone dose (i.e. accepted as equivalent to normal physiological cortisol output) as 4–6 mg once-daily (OD) (12). The guidance provides a choice of two scenarios to guide weaning: i) monitor patients for signs of AI during gradual tapering or ii) routine morning serum cortisol testing. With regard to scenario (i), if patients experience symptoms of AI, tapering should be paused and scenario (ii) initiated. With regard to scenario (ii), if cortisol is >300 nmol/L, then GCs may be stopped safely. If cortisol is 150–300 nmol/L or <150 nmol/L, then this should be repeated in a few weeks or months, respectively, and the current dose continued (12). However, the quality of evidence supporting the ESE/ES recommendation is very low.

The guidance is based in part on the assumption that only ‘supraphysiological’ doses suppress the HPA axis, but this binary model of axis suppression may oversimplify the continuous nature of GC-mediated negative feedback. Furthermore, due to preserved mineralocorticoid function (13), the risk of adrenal crisis is lower in GC-AI than in primary AI. Given that current cortisol thresholds were developed primarily for diagnosing primary and secondary AI, their application to GC-AI may be inappropriate and risk prolonging GC exposure. Individuals with GC-AI have no intrinsic anterior pituitary or adrenal pathology, so they should be expected to achieve HPA axis recovery.

Despite the clinical burden of prolonged GC use, there is a paucity of evidence to guide optimal tapering strategies. We aim to assess how HPA axis function evolves during a physiological taper, the proportion of patients who successfully discontinue prednisolone, how their axis function aligns with guideline thresholds, and the main barriers to successful discontinuation.

## Materials and methods

### Study design and setting

This retrospective cohort study was conducted at a central London teaching hospital. Patients who underwent prednisolone tapering between January 2019 and December 2024 were included, with data collection extending to April 2025 to capture relevant follow-up outcomes. This was a registered retrospective audit of routinely collected clinical data (END_035). Patients were managed by specialist endocrinologists, and tapering was generally guided by the Imperial Centre for Endocrinology (ICE) protocol (14), endorsed by the National Institute for Health and Care Excellence (NICE) (15). The protocol was applied flexibly, with decisions made holistically based primarily on patient-reported symptoms, while also considering dose, HPA axis function, and prednisolone replacement levels (16). Clinicians consistently encouraged full discontinuation of prednisolone. Safety netting measures were routinely implemented, including patient education on sick day rules and the symptoms of AI. While short Synacthen tests (SSTs) were sometimes used to monitor recovery, results were not the sole determinant of tapering decisions, consistent with the holistic approach described above.

### Patients and data collection

**Figure 1:**
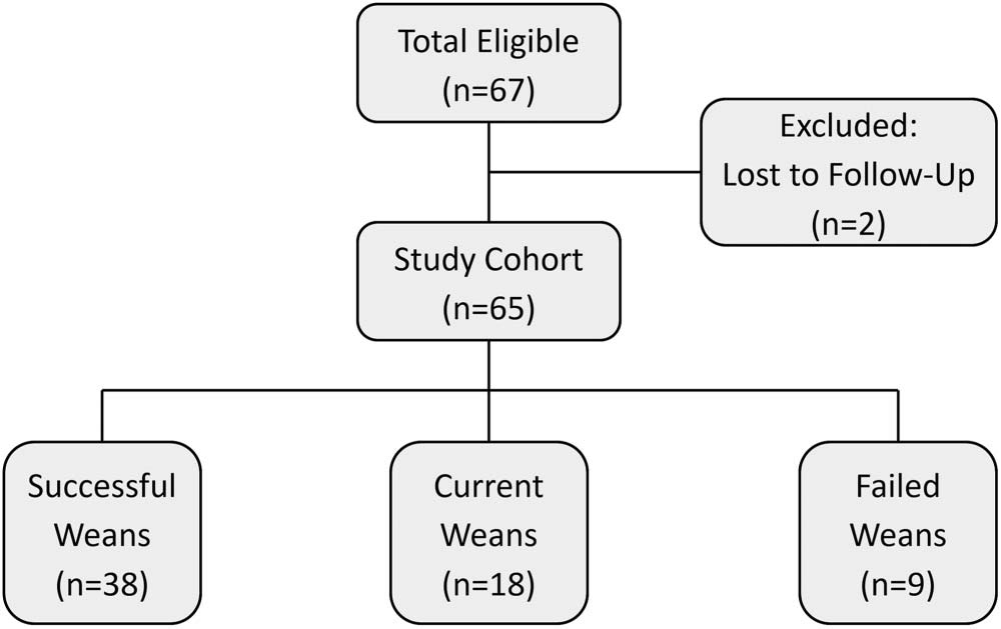
Prednisolone weaning outcome classification in the study cohort. Flowchart illustrating the selection and classification of patients based on weaning status at the time of analysis. Successful weans discontinued prednisolone; current weans were still actively tapering; failed weans remained on prednisolone and were not actively tapering.

Patients were eligible if they were aged 18 years or older, had received at least three months of GC therapy for inflammatory disease, and underwent prednisolone tapering managed by our tertiary endocrine service. Patients were excluded if lost to follow-up (Fig. 1), with available records insufficient to determine weaning outcome. Eligible cases were identified via endocrine multidisciplinary meeting records, and relevant data were extracted from the electronic health record system.

### Definitions

A successful wean was defined as complete discontinuation of prednisolone, with the patient remaining well for at least one month following discontinuation. A failed wean referred to a taper attempt that did not result in full discontinuation, with no further dose reductions being pursued at the time of analysis. A current wean was defined as an ongoing taper, with the patient still actively reducing their dose at the time of analysis.

### SST and laboratory methods

The SST protocol involved intramuscular or intravenous injection of 250 μg tetracosactide, with measurement of ACTH and serum cortisol at baseline and serum cortisol repeated at 30- and 60-min post-injection. Patients were advised to withhold all GC therapy from the preceding evening until the test was completed. Baseline cortisol values were only included if measured between 07:00 and 10:00 h, allowing them to be treated as equivalent to morning cortisol. Peak cortisol was defined as the highest post-injection value. Cortisol was measured using the Abbott Alinity assay and ACTH via the Siemens IMMULITE platform. Prednisolone levels were assessed using ultra-high-performance liquid chromatography coupled with tandem mass spectrometry, with full methodology described previously (17).

### Study outcomes

Serial SSTs at reducing doses conducted at prednisolone doses ≤5 mg were analysed to assess whether HPA axis function improved with tapering at ‘physiological equivalent’ doses. For each successful wean, the lowest morning cortisol measured at doses ≤5 mg was identified to determine how values compared with guideline thresholds. Additional outcomes included the proportion of patients who successfully discontinued prednisolone, time to discontinuation, reasons for weaning failure, incidence of adrenal crises, paired changes in HbA1c, and the prevalence of osteoporosis and fractures. Exploratory analyses were conducted to examine prednisolone pharmacokinetics in patients who struggled to recover HPA axis function.

### Statistical analysis

Statistical analysis was performed using GraphPad Prism (v10.4.2) and IBM SPSS Statistics (v30). A two-tailed *P*-value <0.05 was considered statistically significant. Where presented, confidence intervals (CIs) are reported at the 95% level. Continuous variables were summarised using mean ± standard deviation (SD) if normally distributed, or median with interquartile range (IQR) otherwise. Categorical data were summarised using frequency and percentage. Paired comparisons between two groups with normally distributed differences were assessed using paired *t*-tests, while the Mann–Whitney U test was used for independent non-parametric comparisons of two groups.

Linear mixed-effects models (LMMs) were used to evaluate relationships between prednisolone dose and HPA axis function. Individual dose or dose reduction groups were included as a fixed effect, and patient identity was included as a random effect. We examined serial baseline cortisol values across individual dose reduction. We also evaluated changes in baseline cortisol against grouped magnitudes of dose reduction. In addition, we examined serial peak cortisol values across individual dose reduction. Finally, we evaluated changes in peak cortisol against grouped magnitudes of dose reduction. Model assumptions, including normality of residuals and homoscedasticity, were satisfied. Where applicable, post hoc pairwise comparisons were adjusted using the Bonferroni correction.

## Results

### Baseline characteristics and weaning outcome categorisation

A total of 65 patients were included in the analysis. Baseline characteristics are summarised in Table 1. The mean age at referral was 55.4 ± 16.4 years, and 61.5% (*n* = 40) were female. At referral, the median prednisolone dose was 5 (3.5–5) mg, with the median duration of prednisolone therapy being 23 (6.5–66.5) months. Eighteen patients were actively tapering at the time of analysis. Among the remaining 47 patients, 81% (*n* = 38) successfully weaned (Fig. 1).

**Table 1:** Baseline characteristics of the study cohort at referral (*n* = 65).

Characteristic	Value
Age (mean ± SD)	55.4 ± 16.4 years
Gender, *n* (%)	Female: 40 (61.5%)
	Male: 25 (38.5%)
Ethnicity, *n* (%)	Caucasian: 34 (52.3%)
	Asian: 14 (21.5%)
	Black: 8 (12.3%)
	Other/unknown: 9 (13.8%)
Primary specialty, *n* (%)	Respiratory: 22 (33.8%)
	Rheumatology: 14 (21.5%)
	Gastroenterology: 6 (9.2%)
	Haematology: 6 (9.2%)
	Dermatology: 5 (7.7%)
	Neurology: 5 (7.7%)
	Other: 7 (10.8%)
Inhaled glucocorticoid use, *n* (%)	Yes: 31 (47.7%)
	No: 34 (52.3%)
Topical glucocorticoid use, *n* (%)	Yes: 12 (18.5%)
	No: 53 (81.5%)
Prednisolone dose (median (IQR))	5 (3.5–5) mg
Duration of prednisolone use (median (IQR))	23 (6.5–66.5) months

### Physiological dose tapering promotes HPA axis recovery

**Figure 2:**
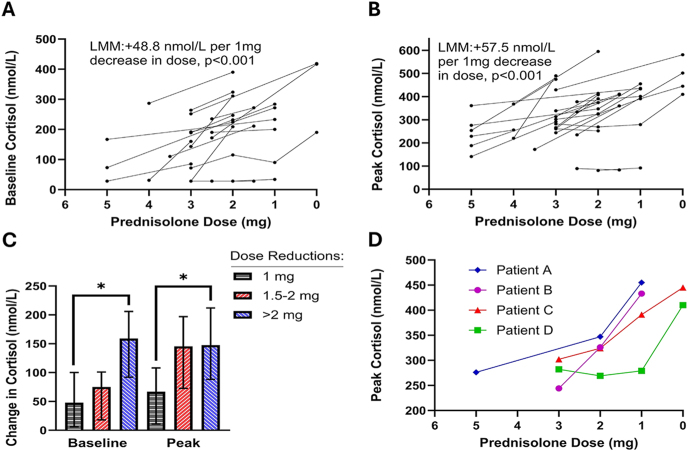
Physiological prednisolone dose tapering promotes HPA axis recovery. Serial short Synacthen tests (SSTs; *n* = 52) were performed at reducing prednisolone doses ≤5 mg in 23 patients. Baseline (A) and peak (B) cortisol values are plotted against reducing dose; each continuous line represents one patient. Linear mixed-effects models (LMMs) were used to assess the association between dose and cortisol values. Cortisol changes were also analysed by magnitude of dose reduction (C). The bars represent medians; the error bars show interquartile ranges. Median dose reductions were 2 mg in the 1.5–2 mg groups and 3 mg in the >2 mg groups. *P < 0.05, determined using LMMs with Bonferroni-corrected post hoc comparisons. Peak cortisol trajectories for patients with ≥3 SSTs at reducing doses ≤5 mg are shown individually (D).

Twenty-three patients undergoing a prednisolone taper underwent more than one SST. Two patients had four SSTs, another two had three SSTs, and 19 underwent two SSTs over the weaning period (Fig. 2). LMMs showed a significant positive association between reducing prednisolone dose and improvements in HPA axis function. For each 1 mg reduction in dose, baseline cortisol increased by an estimated 48.8 nmol/L (CI: 30.6–67.0, *P* < 0.001; Fig. 2A), and peak cortisol increased by 57.5 nmol/L (CI: 39.5–75.5, *P* < 0.001; Fig. 2B). Grouping changes in SST results by magnitude of dose reduction (1 mg, 1.5–2 mg, >2 mg), LMMs identified significant interactions with cortisol changes for both baseline (*P* = 0.018) and peak (*P* = 0.010) cortisol (Fig. 2C). Post hoc comparisons revealed greater increases following >2 mg reductions compared to 1 mg reductions for both baseline (*P* = 0.013) and peak (*P* = 0.022) cortisol. Individual patient trajectories of peak cortisol for those with ≥3 SSTs at reducing doses are shown (Fig. 2D). Of note, patient D had adequate 8 h prednisolone replacement levels on 2 mg, suggesting that this dose constituted full ‘physiological equivalent’ replacement.

### Successful weans

**Figure 3:**
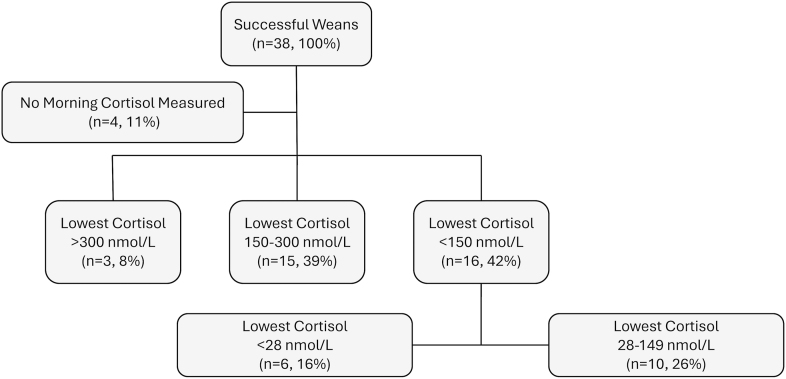
Even undetectable cortisol values did not preclude successful weaning. Lowest recorded morning cortisol values at prednisolone doses ≤5 mg for each of the 38 successful weans were analysed. Values were obtained from either standard morning cortisol measurements or baseline values from short Synacthen tests. Cortisol levels <28 nmol/L were below the assay’s detection limit.

Among those who discontinued prednisolone (*n* = 38), 8% (*n* = 3) had their lowest recorded morning cortisol >300 nmol/L (Fig. 3). A further 39% (*n* = 15) had lowest values between 150 and 300 nmol/L, and 42% (*n* = 16) had values <150 nmol/L. Notably, 16% (*n* = 6) had undetectable levels (<28 nmol/L). The median time to discontinuation was 8 (5–15.3) months. No adrenal crises, deaths, or GC reinstatement for AI occurred post-discontinuation during the available follow-up period. In 18% (*n* = 7), the wean included a temporary prednisolone increase above 5 mg due to relapse of the primary condition, but patients ultimately achieved full discontinuation.

### Barriers to weaning

Of the nine patients who failed to wean, reasons included uncontrolled inflammatory disease (*n* = 5), insufficient HPA axis recovery (*n* = 3), and intolerable withdrawal symptoms despite normal HPA axis function (*n* = 1). No adrenal crises occurred across the cohort during the weaning process.

All SSTs (*n* = 10) in the three patients who failed to wean due to insufficient HPA axis recovery were performed at prednisolone doses <3 mg and showed a consistent triad: ACTH ≤10.7 ng/L, baseline cortisol ≤34 nmol/L, and peak cortisol ≤92 nmol/L. Despite this, these patients remained well on a maintenance dose of 2 mg (*n* = 2) or 1 mg (*n* = 1). All three had adequate 8 h prednisolone replacement levels on 2 mg.

A fourth patient with similar results on 1 mg prednisolone (ACTH <5 ng/L; baseline <28 nmol/L; peak 59 nmol/L) also had adequate 8 h prednisolone replacement levels on 2 mg, suggesting that they were a slow GC metaboliser. Therefore, they were switched to hydrocortisone OD, which has a shorter duration of action than prednisolone. Following this, they were able to successfully wean, and on 5 mg hydrocortisone OD, a repeat SST showed substantial recovery (ACTH 45.6 ng/L; baseline 111 nmol/L; peak 125 nmol/L).

### GC-related morbidity

GC-associated adverse effects were common. Overall, 48% (*n* = 31) had either osteopenia or osteoporosis, with 23% (*n* = 15) having osteoporosis and 22% (*n* = 14) having experienced a fracture. Vitamin D, calcium, and bisphosphonates were prescribed in 91% (*n* = 59), 58% (*n* = 38), and 35% (*n* = 23), respectively. Dysglycaemia was also frequent, with 34% (*n* = 22) having prediabetes or T2DM, including 15% (*n* = 10) with T2DM.

**Figure 4:**
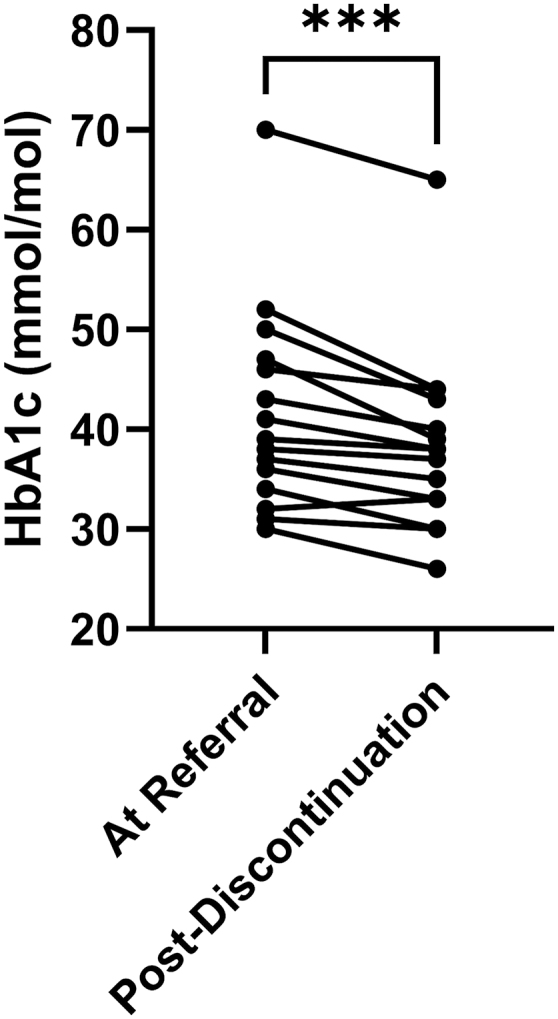
HbA1c significantly improves following prednisolone discontinuation. Paired HbA1c measurements from 15 patients were compared at the time of endocrine referral and following successful discontinuation of prednisolone. The median prednisolone dose at referral was 5 (4–5) mg. Mean HbA1c decreased from 41.7 mmol/mol at referral to 38.3 mmol/mol after discontinuation. ***P < 0.001, determined using a paired *t* test.

Among patients with paired HbA1c values at referral and post-discontinuation (*n* = 15), there was a significant reduction following discontinuation (mean difference: −3.4 mmol/mol, *P* = 0.0002; Fig. 4). The median referral dose was 5 (4–5) mg. When restricted to patients with baseline HbA1c ≥42 mmol/mol (*n* = 6), the reduction was greater (mean difference: −5.5 mmol/mol, *P* = 0.0035). The median referral dose in this subgroup did not differ significantly from that of patients outside the subgroup (5 vs 5 mg, *P* = 0.93).

## Discussion

This study aimed to evaluate real-world outcomes of prednisolone weaning at a specialist endocrine centre, focusing on how HPA axis function evolves during physiological dose reduction. Our findings demonstrate that HPA axis function is dynamic, improving with tapering even starting at or below ‘physiological equivalent’ doses and that current guidelines are overly conservative, restricting successful weaning. Together, these findings have significant implications for the management of GC-AI, challenging both the rationale and clinical utility of existing cortisol threshold-based guidance.

Our data challenge the assumption that only ‘supraphysiological’ GC doses suppress the HPA axis. We observed an estimated increase in morning and peak cortisol of 48.8 nmol/L and 57.5 nmol/L, respectively, for each milligram reduction in prednisolone, with larger dose reductions producing greater cortisol increases. These findings align with the principle that the negative feedback from exogenous GCs on the HPA axis is a continuum and will depend on the residual exogenous GC present, most critically during the early morning, when ACTH pulsatility increases to trigger the morning rise in cortisol secretion. A recent study reported that patients starting their wean from 3 mg still experienced significant rises in morning cortisol following discontinuation (18), indicating that even at these low end ‘physiological equivalent’ doses, there is a significant repressive effect on the HPA axis. These findings suggest that HPA axis recovery is driven by dose reduction itself, with successful tapering enabling rather than following axis recovery.

Our holistic approach likely contributed to the high successful weaning rate of 81%, allowing tapering to continue even in patients starting with extremely low morning cortisol values. In 42% of successful weans, the lowest recorded morning cortisol was below 150 nmol/L, and in several cases, it was undetectable. Tapering was tolerated and ultimately successful. This contradicts current guidance, which interprets such values as evidence of persistent AI (12) requiring long-term GC replacement, implying that tapering from this position might be futile. These results highlight the inadequacy of fixed cortisol thresholds in GC-AI and support a strategy focused on symptom-led tapering with appropriate safety-netting.

Notably, no adrenal crises occurred during the weaning process, highlighting the safety of this approach. The average dose at referral was 5 mg, which likely reflects a reluctance among referring clinicians to taper below this level, shaped by current guidelines and concern over adrenal crisis risk. Recent studies support our findings that it is safe to wean without confirming complete HPA axis recovery and that recovery of the axis occurs with cessation of GCs (18, 19, 20). The normal aldosterone axis in GC-AI likely contributes to the reduced adrenal crisis risk. Unnecessarily continuing patients on currently defined ‘physiological equivalent’ doses increases the risk of GC-related morbidity, including severe infections and sepsis (8), and mortality (6). Aiming for complete discontinuation must therefore be a consistent priority.

The median time to discontinuation was eight months, broadly aligning with the 24-week ICE protocol (14). This timeline reflects the protocol’s use as a flexible framework rather than a rigid schedule, with pauses during tapering for symptom management or inflammatory disease relapse. Evidence comparing GC tapering durations is limited (18, 21, 22). A rapid tapering schedule after short-term GC use for severe COVID-19 infection has been reported with good recovery of HPA axis function (23). Future research should assess whether tapering schedules shorter than the 24-week ICE protocol can safely support HPA axis recovery following long-term GC therapy. Importantly, implementation of physiological dose tapering may vary between countries depending on the availability of low-dose prednisolone, which may limit the use of 1 mg dose reductions in some settings.

Perseverance during the taper was often critical: 18% of successful weans required temporary increases in prednisolone dose to manage inflammatory disease flare-ups before ultimately discontinuing treatment. This demonstrates the importance of continued collaboration with the primary specialty, as tapering may remain feasible even after temporary setbacks. While inflammatory disease teams have traditionally employed slow weaning protocols to mitigate disease relapse risk (21), the availability and efficacy of biologic therapies have enabled more rapid GC tapers down to ‘physiological equivalent’ doses. This may result in lower morning cortisol levels at the point these doses are reached, further highlighting the need to move away from cortisol threshold-based guidance. Importantly, no cortisol value, however low, should in itself preclude continued weaning.

One patient was unable to wean due to GC withdrawal symptoms despite normal HPA axis function. Although infrequent in our cohort, GC withdrawal syndrome is a recognised barrier to tapering (24) and warrants further study to guide optimal management. Importantly, patients classified as failed weans remain under follow-up, and this status reflects their position at the time of analysis rather than a permanent outcome.

Our findings also highlight the role of pharmacokinetic variability in persistent HPA axis suppression. All three patients who failed to wean due to insufficient axis recovery had adequate 8 h prednisolone levels on 2 mg, suggesting this dose constituted full ‘physiological equivalent’ replacement in those individuals. This challenges existing definitions of a ‘physiological equivalent’ dose (ESE/ES 4–6 mg (12); NICE 3–5 mg (15)). Slow GC metabolism may enhance the suppressive effect of low doses, with even 1 mg prednisolone sufficient to perpetuate HPA axis suppression in some patients. This pattern was evident in the trajectory of patient D (Fig. 2D), whose cortisol levels only began to rise after complete prednisolone discontinuation. Another patient with this profile successfully weaned after switching to hydrocortisone OD, which has a shorter duration of action and lower GC receptor potency than prednisolone (25), resulting in lower cumulative GC exposure. The marked improvement in HPA axis function following this switch supports the idea that even 1 mg prednisolone can provide enough negative HPA axis feedback to be fully suppressive. It has previously been demonstrated that lower doses such as 2 mg prednisolone should be considered ‘physiological equivalent’ in some patients (16), and these findings provide further support for this notion.

GC-related morbidity was common. 48% of the cohort had osteopenia or osteoporosis, and 22% had sustained a fracture. Dysglycaemia was also prevalent, with 34% having prediabetes or T2DM. Among those with baseline prediabetes or T2DM, discontinuation of prednisolone led to a mean HbA1c reduction of 5.5 mmol/mol. These findings reinforce the importance of discontinuing GCs where possible and suggest that preventative measures, including careful monitoring and early intervention, may be warranted in patients with prediabetes or T2DM commencing GC therapy.

**Figure 5:**
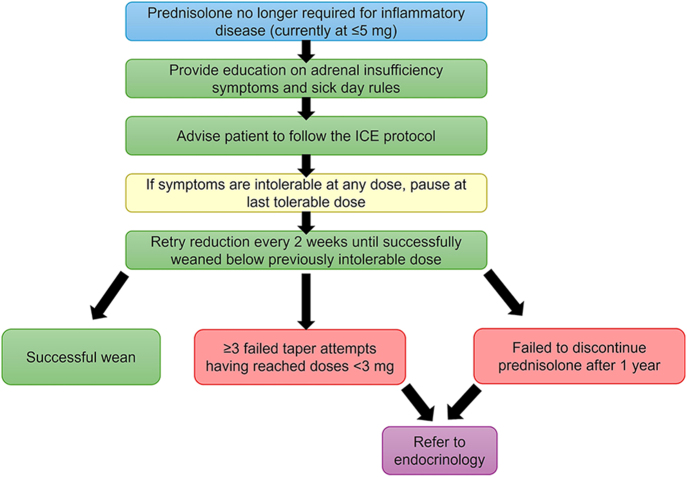
Proposed clinical pathway for prednisolone weaning in GC-AI. Schematic overview of a proposed tapering strategy for patients at risk of glucocorticoid-induced adrenal insufficiency (GC-AI) who no longer require prednisolone for their underlying inflammatory condition. The approach incorporates the Imperial Centre for Endocrinology (ICE) protocol (14) with symptom-led dose reduction and criteria for endocrine referral.

Drawing on our results and clinical experience, we propose a structured framework for the management of GC-AI (Fig. 5). This includes practical guidance on symptom-led tapering and endocrine referral criteria for clinicians. We recommend prioritising tapering over cortisol thresholds, avoiding routine HPA axis testing during the wean, and encouraging persistence supported by safety-netting. We recommend against routine post-discontinuation HPA axis assessment, supported by a recent study highlighting the low GC-AI prevalence following a successful wean (19). Even if morning cortisol does not reach the guideline threshold in an asymptomatic patient, we would not recommend restarting daily GC replacement because these patients have adequate levels of aldosterone. Where clinical concern exists, continued sick-day rule cover provides sufficient safety while avoiding negative feedback from daily replacement that could impair HPA axis recovery. While our recommendations are similar to scenario (i) from the ESE/ES guidance (12), a key difference is that we recommend avoiding HPA axis assessment even following the development of AI symptoms, instead pausing briefly on a comfortable dose before attempting another taper. Mild symptoms are to be expected during tapering, with controlled physiological stress from ongoing dose reduction acting as the stimulus that promotes HPA axis recovery.

This study has several strengths. It provides clear evidence that HPA axis function improves with prednisolone dose reduction, even at physiological doses, challenging the validity of existing cortisol threshold-based guidance. It offers novel pharmacokinetic insights and presents real-world outcomes using individual-level longitudinal data. The cohort was ethnically diverse and included referrals from a broad range of specialties, supporting the generalisability of findings across different patient groups and clinical contexts. Limitations include the retrospective design, potential selection bias from being conducted at a specialist centre, variability in clinical decision-making, and a modest cohort size, with small subgroup sizes for certain analyses.

Further prospective research is needed to validate these findings. A multicentre cohort study using a standardised weaning protocol with predefined HPA axis testing points would improve generalisability and precision. Future work should incorporate objective assessment of symptoms, quality of life, and additional parameters of adrenal insufficiency, including sodium, glucose, and blood pressure, during and after tapering. Additional further work should evaluate whether salivary cortisone or cortisol may offer a suitable and less invasive alternative to serum cortisol for monitoring recovery in GC-AI. Standardised follow-up after GC discontinuation is warranted to further evaluate the long-term safety of symptom-led tapering. Additional work on GC pharmacokinetics may help identify patients at risk of prolonged suppression and determine whether hydrocortisone OD switching can reliably facilitate axis recovery in selected cases.

In conclusion, prolonged GC therapy carries significant risks, with weaning hindered by the limitations of current guidance. We have shown that HPA axis function in GC-AI improves with dose reduction, even at physiological doses, and that structured, symptom-led tapering supported by safety-netting is both safe and effective.

## Declaration of interest

The authors declare that there is no conflict of interest that could be perceived as prejudicing the impartiality of the work reported.

## Funding

The Section of Endocrinology and Investigative Medicine was funded by grants from the UK Medical Research Council, Biotechnology and Biological Sciences Research Council, and NIHR. KL is a Diabetes UK Sir George Alberti Research Training Fellow (grant reference number 23/0006515). The open access fee was paid by the Imperial College Open Access Fund.
